# Socioeconomic inequality in the worsening of psychosocial wellbeing *via* disrupted social conditions during COVID-19 among adolescents in Hong Kong: self-resilience matters

**DOI:** 10.3389/fpubh.2023.1136744

**Published:** 2023-04-26

**Authors:** Gary Ka-Ki Chung, Yat-Hang Chan, Thomas Sze-Kit Lee, Siu-Ming Chan, Ji-Kang Chen, Hung Wong, Roger Yat-Nork Chung, Esther Sui-Chu Ho

**Affiliations:** ^1^CUHK Institute of Health Equity, The Chinese University of Hong Kong, Shatin, Hong Kong SAR, China; ^2^JC School of Public Health and Primary Care, The Chinese University of Hong Kong, Shatin, Hong Kong SAR, China; ^3^Department of Educational Administration and Policy, Faculty of Education, The Chinese University of Hong Kong, Shatin, Hong Kong SAR, China; ^4^Department of Social and Behavioural Sciences, The City University of Hong Kong, Kowloon, Hong Kong SAR, China; ^5^Department of Social Work, The Chinese University of Hong Kong, Shatin, Hong Kong SAR, China; ^6^CUHK Centre for Bioethics, The Chinese University of Hong Kong, Shatin, Hong Kong SAR, China

**Keywords:** adolescents, COVID-19, psychosocial wellbeing, resilience, socioeconomic inequalities

## Abstract

**Background:**

Adolescents, especially the socioeconomically disadvantaged, are facing devastating psychosocial impact of the COVID-19 pandemic during their critical developmental period. This study aims to (i) examine the socioeconomic patterning of the worsening of psychosocial wellbeing, (ii) delineate the underlying mediating factors (i.e., overall worry about COVID-19, family's financial difficulty, learning problems, and loneliness), and (iii) explore the moderating effect of resilience in the inter-relationship among adolescents under COVID-19.

**Methods:**

Based on maximum variation sampling of 12 secondary schools of diverse socioeconomic background in Hong Kong, 1018 students aged 14-16 years were recruited and completed the online survey between September and October 2021. Multi-group structural equation modeling (SEM) by resilience levels was employed to delineate the pathways between socioeconomic position and the worsening of psychosocial wellbeing.

**Results:**

SEM analysis showed a significant total effect of socioeconomic ladder with the worsening of psychosocial wellbeing during the pandemic in the overall sample (β = −0.149 [95% CI = −0.217 – −0.081], *p* < 0.001), which operated indirectly through learning problems and loneliness (both *p* < 0.001 for their indirect effects). Consistent pattern with stronger effect size was observed in the lower resilience group; nonetheless, the associations were substantially mitigated in the higher resilience group.

**Conclusion:**

In addition to facilitating self-directed learning and easing loneliness during the pandemic, evidence-based strategies to build up resilience among adolescents are critical to buffer against the adverse socioeconomic and psychosocial impacts of the pandemic or other potential catastrophic events in the future.

## Introduction

With the emergence of new variants of concern, the COVID-19 pandemic continues to spread across the globe. Apart from the significant disease burden and far-reaching economic consequences, an extensive body of evidence suggests that the pandemic has exposed and amplified the underlying social inequalities in societies. In addition to the higher incidence and mortality in the disadvantaged communities (1) the broader impact of the pandemic on social determinants of health and the associated health inequalities have also been widely observed, (2) even in regions such as Hong Kong with relatively less severe outbreak due to the differential impact of the mandatory COVID-19 containment measures across the socioeconomic ladder (3–6).

In particular, adolescents are facing detrimental psychosocial impact of the COVID-19 pandemic during their critical developmental period (7, 8) especially for the socioeconomically disadvantaged (9, 10) Under the ongoing epidemic, prolonged school closure and stringent social distancing policies exacerbated the psychosocial wellbeing of adolescents and a range of social conditions such as learning opportunities (11, 12) social relationships and connectedness,(13, 14) as well as worries on the pandemic and sense of financial insecurity (15, 16). While most existing studies focus on one specific type of these social conditions, few examined the full picture on how different social conditions during the pandemic are socioeconomically patterned and hence disproportionately worsen the psychosocial wellbeing of adolescents. To inform policy entry points for interventions to mitigate the socioeconomic inequalities in psychosocial wellbeing during the pandemic, it is indispensable to identify the social conditions that are most severely affected by the pandemic among adolescents across the socioeconomic ladder.

Despite the well-documented evidence on the inequitable psychosocial impact of COVID-19, the potential heterogeneity in the socioeconomic patterning of psychosocial wellbeing deserves further investigation into why some adolescents, even if of similar socioeconomic background, have fared better than the others in response to COVID-19. Notably, as highlighted by Dvorsky et al. (17) the resilience of adolescents plays a crucial role in mitigating or even evading the social and mental health challenges under the pandemic, where a higher level of self-resilience facilitates successful adaptation, coping, and recovery in the context of the COVID-19-induced psychosocial distress. While existing COVID-19 studies support the protective effect of resilience on psychosocial wellbeing and its effect modification on certain psychosocial risk factors (18–21), whether resilience status could buffer the impacts of socioeconomic position on psychosocial wellbeing and its determinants remains understudied.

In light of the aforementioned knowledge gaps, the present study aims to (i) assess the association between socioeconomic position and the worsening of psychological wellbeing among adolescents, (ii) delineate how different psychosocial determinants disrupted by the pandemic mediate any observed association between socioeconomic position and the worsening of psychological wellbeing, and (iii) explore the potential moderating effect of resilience on the associations and mediating roles.

## Methods

### Study population

Data were collected from a purposive sample of 12 secondary schools of different socioeconomic background (see the socioeconomic classifications in Supplementary Table 1) in Hong Kong *via* online survey between September and October 2021 (22). Invitation letters were sent to members of the Hong Kong Association of the Heads of Secondary Schools (established by dedicated secondary school principals with a vision to enhance professionalism and the understanding of education in secondary schools) to recruit all Secondary 3 students enrolled to each participating school (equivalent to Grade 9 in the United States or Year 10 in the United Kingdom). Among the 1,467 enrolled Secondary 3 students, 1,254 students were successfully surveyed with a response rate of 85.48%. According to the pre-determined inclusion criteria on age range, 1,095 students aged 14–16 years who consented to participate were eligible for this study. After excluding 77 students with incomplete responses, 1,018 students were included for analysis.

### Measurements

Information on respondents' self-perceived socioeconomic ladder, psychosocial wellbeing and related determinants during COVID-19, resilience status, as well as other socio-demographic and health factors were collected for analyses, with details listed below.

#### Socioeconomic ladder

The self-perceived family's socioeconomic position of respondents was measured using the social ladder measure of the MacArthur Scale of Subjective Social Status – Youth Version (23). Respondents was asked to mark the rung that best represents where their family would be on a socioeconomic ladder ranging from rung 1 (the worst off) to rung 10 (the best off) on a 10-point Likert scale. The MacArthur Scale of Subjective Social Status – Youth Version was adopted as a previous systematic review showed that it is most strongly associated with health outcomes related to psychological processes (24) whereas, previous studies also showed its superior role over objective socioeconomic measures in predicting health outcomes such as self-rated health, depression, and wellbeing among adolescents (24). The socioeconomic ladder was re-categorized into six groups according to the reported score (i.e., ≤3, 4, 5, 6, 7, and ≥8) for analysis.

#### Worsening of psychosocial wellbeing

To assess the change in psychosocial wellbeing, respondents were asked how much more/less they have felt during the pandemic when compared with the time before COVID-19 in terms of (i) relaxed, (ii) confident about future, (iii) cheerful, (iv) anxious/stressed, and (v) hopeless with five ordinal options (i.e., 1= much less; 2 = somewhat less; 3 = about the same; 4 = somewhat more; 5 = much more), which were adopted and modified from the COVID-19 Adolescent Symptom & Psychological Experience Questionnaire. (25) The five selected items captured both positive and negative emotions for a more comprehensive assessment because psychosocial wellbeing refers not only to a high level of positive affect but also a low level of negative affect (26). The latent construct on the ‘worsening of psychosocial wellbeing' was created based on these five items, of which the first three positively worded items were reversely coded for analysis to consistently show the results in one direction.

#### Psychosocial determinants

Four domains of psychosocial determinants during COVID-19 were analyzed as potential mediators of the association between socioeconomic ladder and the worsening of psychosocial wellbeing, which included (i) overall worry about COVID-19, (ii) family's financial difficulty, (iii) learning problems, and (iv) loneliness.

The first two domains were measured using single-item questions. Regarding overall worry about COVID-19, respondents were asked how worried they were about the local COVID-19 situation with five ordinal options (i.e., 1 = not at all; 2 = slightly; 3 = moderately; 4 = very; 5 = extremely). As for family's financial difficulty, respondents were asked to what extent the changes related to the COVID-19 outbreak have created financial problems for their family with five ordinal options (i.e., 1 = not at all; 2 = slightly; 3 = moderately; 4 = very; 5 = extremely). The latter two domains were measured as latent constructs. Regarding learning problems, respondents were asked to what extent they experienced the following problems including (i) internet access, (ii) finding a quiet place to study, (iii) understanding my school assignments, and (iv) finding someone who could help me with my school work, each with four ordinal options (i.e., 1 = never; 2 = sometimes; 3 = often; 4 = always). Loneliness was measured using the UCLA 3-item loneliness scale (27) on (i) feeling that you lack companionship, (ii) feeling left out, and (iii) feeling isolated from others, each with three ordinal options (i.e., 1= hardly ever; 2 = some of the time; 3 = often).

#### Resilience

As a potential moderator for stratified analyses, resilience was measured using the 6-item Brief Resilience Scale which assesses the ability to bounce back or recover from adversities and to cope with health-related stressors (28). Responses were rated on a 5-point Likert scale from “strongly disagree” to “strongly agree” with a possible average score ranging from 1 to 5. The score was then divided into the “higher resilience” and “lower resilience” groups using the sample mean score as the cut-off to ensure similar sample size between the two resilience groups.

### Statistical analysis

Descriptive statistics of respondents were derived using mean with standard deviations (SD) for continuous variables and count with percentages for categorical variables. Confirmatory factor analyses and reliability tests were performed for the latent constructs (i.e., worsening of psychosocial wellbeing, learning problems, and loneliness) to ensure that each of these latent constructs was well-explained by its corresponding observed variables. The minimum acceptable factor loading of the observed variables is 0.30 (29). Separate correlation matrices of the aforementioned variables and constructs were derived for the overall sample, lower resilience group, and higher resilience group.

The inter-relationship among socioeconomic ladder, psychosocial determinants, and the worsening of psychosocial wellbeing during COVID-19 was examined using structural equation modeling (SEM), with adjustments for gender, household size (i.e., six groups ranging from “1” to “6 or above”), and baseline self-reported health status (i.e., a retrospective recall of health status before COVID-19 based on a five-point scale ranging from “poor” to “excellent”). In addition, multi-group SEM analysis was employed to assess the potential heterogeneity of the inter-relationship across the lower and higher resilience groups, which was tested based on the χ^2^ difference between the unconstrained model and structural weight model (i.e., assuming all the paths are equal between the two resilience groups).

We obtained the regression weights of variables as well as the direct and indirect effects on the endogenous variables. Since there are multiple potential mediators in the SEM model, covariance was specified in each of the possible pairs so that the resulted indirect effect of each mediator would be adjusted for the effects of all other mediators. Bootstrapping of 2000 samples and 95% bias-corrected confidence level (CI) were used to estimate the indirect paths. We also estimated the goodness-of-fit of the SEM model, where a root mean square error of approximation (RMSEA) value below 0.08 is deemed having a good model fit (30). Other goodness-of-fit indices, including comparative fit index (CFI), incremental fit index (IFI), and Tucker-Lewis index (TLI), are considered to be satisfactory if they are above 0.90 (31) and superior if they are above 0.95. (30) The adjusted GFI (AGFI) are considered acceptable if the value is above 0.90 (30, 31). SPSS and AMOS version 26 were employed for statistical analyses. All statistical tests were two-tailed with a significant level of 0.05 unless specified.

## Results

Table 1 shows the basic characteristics of our 1018 sampled secondary school students aged 14–16 years (54.0% female). Based on the 10-rung socioeconomic ladder, the respective proportions of those who rated ≤3, 4, 5, 6, 7, and ≥8 were 11.0, 13.4, 31.8, 20.5, 13.1, and 10.2%. Regarding the change in psychosocial wellbeing, 22.6% felt less relaxed, 32.6% felt less confident about the future, 21.6% felt less cheerful, 35.6% felt more anxious or stressful, and 17.1% felt more hopeless during COVID-19. Descriptive statistics on resilience, overall worry about COVID-19, family's financial difficulty, learning problems, loneliness, and other demographic factors and health status are also reported in Table 1.

**Table 1:** Basic characteristics of respondents (*n* = 1018).

	***N* (%) or Mean ±SD**
**Resilience**	3.14 ± 0.69
**Gender**	
Female	550 (54.0)
Male	468 (46.0)
**Household size**	
1	15 (1.5)
2	60 (5.9)
3	228 (22.4)
4	394 (38.7)
5	214 (21.0)
6 or above	107 (10.5)
**Baseline health status**	
Poor	34 (3.3)
Fair	258 (25.3)
Good	345 (33.9)
Very good	202 (19.8)
Excellent	179 (17.6)
**Socioeconomic ladder (10-rung)**	
3 or below	112 (11.0)
4	136 (13.4)
5	324 (31.8)
6	209 (20.5)
7	133 (13.1)
8 or above	104 (10.2)
**Overall worry about COVID-19**	
Not at all	183 (18.0)
Slightly	395 (38.8)
Moderately	310 (30.5)
Very	86 (8.4)
Extremely	44 (4.3)
**Family's financial difficulty**	
Not at all	260 (25.5)
Slightly	351 (34.5)
Moderately	312 (30.6)
Very	74 (7.3)
Extremely	21 (2.1)
**Learning problems**	
Internet access	
Never	642 (63.1)
Sometimes	310 (30.5)
Often	45 (4.4)
Always	21 (2.1)
Finding a quiet place to study	
Never	473 (46.5)
Sometimes	369 (36.2)
Often	122 (12.0)
Always	54 (5.3)
Understanding my school assignments	
Never	332 (32.6)
Sometimes	490 (48.1)
Often	128 (12.6)
Always	68 (6.7)
Finding someone who could help me with my school work	
Never	464 (45.6)
Sometimes	390 (38.3)
Often	100 (9.8)
Always	64 (6.3)
**Loneliness**	
Feeling that you lack companionship	
Hardly ever	563 (55.3)
Some of the time	317 (31.1)
Often	138 (13.6)
Feeling left out	
Hardly ever	629 (61.8)
Some of the time	272 (26.7)
Often	117 (11.5)
Feeling isolated from others	
Hardly ever	680 (66.8)
Some of the time	243 (23.9)
Often	95 (9.3)
**Change in psychosocial wellbeing**	
Relaxed	
Much less	82 (8.1)
Somewhat less	148 (14.5)
About the same	527 (51.8)
Somewhat more	170 (16.7)
Much more	91 (8.9)
Confident about the future	
Much less	97 (9.5)
Somewhat less	235 (23.1)
About the same	523 (51.4)
Somewhat more	110 (10.8)
Much more	53 (5.2)
Cheerful	
Much less	69 (6.8)
Somewhat less	151 (14.8)
About the same	497 (48.8)
Somewhat more	206 (20.2)
Much more	95 (9.3)
Anxious/stressed	
Much less	92 (9.0)
Somewhat less	124 (12.2)
About the same	440 (43.2)
Somewhat more	270 (26.5)
Much more	92 (9.0)
Hopeless	
Much less	159 (15.6)
Somewhat less	125 (12.3)
About the same	560 (55.0)
Somewhat more	129 (12.7)
Much more	45 (4.4)

Table 2 presents the standardized factor loadings of the three latent constructs, which ranged from 0.463 to 0.865 for worsening of psychosocial wellbeing (covariance between the last two items was specified as they were negatively worded), from 0.429 to 0.794 for learning problems, and from 0.770 to 0.961 for loneliness. Acceptable reliability was observed for the three latent constructs (Cronbach's alpha = 0.774, 0.745, and 0.896, respectively).

**Table 2:** Standardized factor loadings of observed variables on latent constructs based on separate confirmatory factor analyses.

**Latent construct**	**Observed variables**	**Factor loading**
Worsening of psychosocial wellbeing (Cronbach's alpha = 0.774)	*Compared with the time before the COVID-19 pandemic, how much more/less have you felt in the following ways during the COVID-19 pandemic?*	
	1. Relaxed	0.603
	2. Confident about the future	0.612
	3. Cheerful	0.865
	4. Anxious/stressed	0.463
	5. Hopeless	0.513
Learning problems	*During the time when your school was closed because of COVID-19, how often did you have the following problems when completing your school work?*	
(Cronbach's alpha = 0.745)	1. Problems with Internet access	0.429
	2. Problems with finding a quiet place to study	0.592
	3. Problems with understanding my school assignments	0.794
	4. Problems with finding someone who could help me with my school work	0.768
Loneliness	*Please indicate how often each of the statements below is descriptive of you during the COVID-19 pandemic*.	
(Cronbach's alpha = 0.896)	1. Feeling that you lack companionship	0.770
	2. Feeling left out	0.961
	3. Feeling isolated from others	0.861

**Figure 1:**
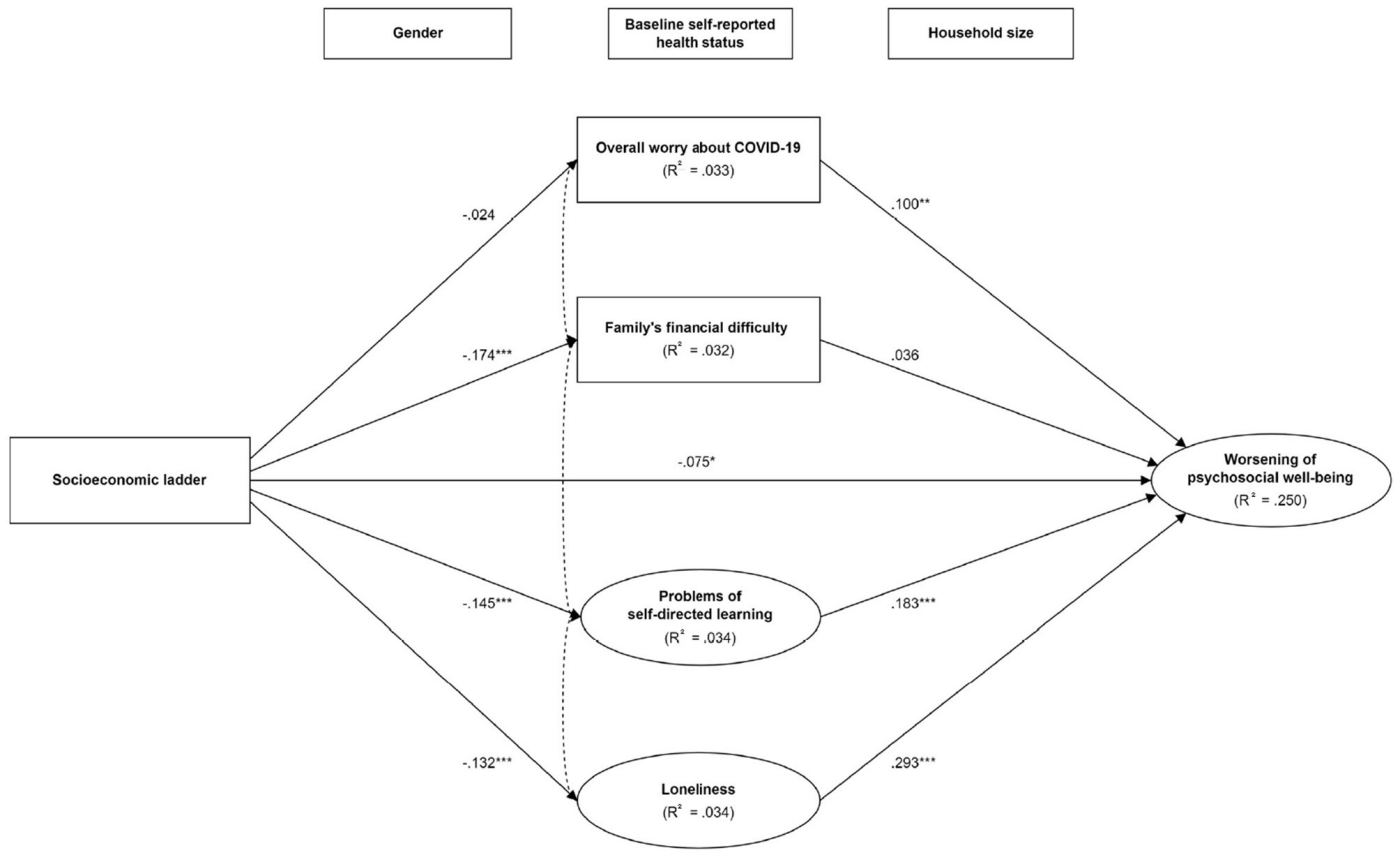
The mediating pathways between socioeconomic position and the worsening of psychosocial wellbeing in the overall sample. ^*^p < 0.05; ^**^p < 0.01; ^***^p < 0.001. Model was adjusted for gender, household size, and baseline self-reported health status. Covariance was specified in each of the possible pairs of mediators. The dotted double arrows between mediators are simplified for better readability.

Table 3 displays the correlation matrices of all variables and constructs in the overall sample, lower resilience group, and higher resilience group. The resultant SEM model on the overall sample yielded satisfactory model fit to the data, with χ^2^ (df = 104, *N* = 1018) = 344.517, *p* < 0.001, RMSEA = 0.048, RMR = 0.032 CFI = 0.954, IFI = 0.955, TLI = 0.933, and AGFI = 0.941, suggesting a satisfactory model fit. After adjustment for gender, household size, and baseline health status, significant total effect between socioeconomic ladder and the worsening of psychosocial wellbeing due to COVID-19 was observed (β = −0.149 [95% CI = −0.217 – −0.081], *p* < 0.001). As shown in Figure 1, significant direct effects of the socioeconomic ladder were observed with the worsening of psychosocial wellbeing, family's financial difficulty, learning problems, and loneliness during COVID-19, whereas loneliness, learning problems, and overall worry about COVID-19 were significant predictors of the worsening of psychosocial wellbeing. Specifically, the socioeconomic patterning of the worsening of psychosocial wellbeing operated indirectly through learning problems (*p* < 0.001) and loneliness (*p* < 0.001).

**Table 3:** Correlation matrix among observed variables.

			**1**	**2**	**3**	**4**	**5**	**6**	**7**	**8**	**9**
**Overall sample (*****n** =* **1018)**											
	1	Socioeconomic ladder	1								
	2	Overall worry about COVID-19	−0.021	1							
	3	Family's financial difficulty	−0.170^***^	0.237^***^	1						
	4	Learning problems	−0.150^***^	0.187^***^	0.267^***^	1					
	5	Loneliness	−0.149^***^	0.120^***^	0.177^***^	0.472^***^	1				
	6	Worsening of psychosocial wellbeing	−0.169^***^	0.182^***^	0.175^***^	0.373^***^	0.423^***^	1			
	7	Gender	−0.048	0.157^***^	0.046	0.096^**^	0.076^*^	0.125^**^	1		
	8	Household size	0.091^**^	−0.034	−0.007	0.038	−0.016	0.025	0.004	1	
	9	Baseline health status	0.159^**^	0.073^*^	0.003	−0.059	−0.109^***^	−0.143^***^	−0.042	−0.020	1
**Lower resilience group (*****n** =* **549; 53.9%)**											
	1	Socioeconomic ladder	1								
	2	Overall worry about COVID-19	0.010	1							
	3	Family's financial difficulty	−0.139^**^	0.275^***^	1						
	4	Learning problems	−0.158^***^	0.170^***^	0.212^***^	1					
	5	Loneliness	−0.139^**^	0.137^**^	0.170^***^	0.467^***^	1				
	6	Worsening of psychosocial wellbeing	−0.162^***^	0.210^***^	0.166^**^	0.405^***^	0.472^***^	1			
	7	Gender	0.031	0.096^*^	0.033	0.083	0.096^*^	0.067	1		
	8	Household size	0.103^*^	−0.039	0.015	−0.009	−0.002	0.033	0.009	1	
	9	Baseline health status	0.082	0.118^**^	0.045	−0.013	−0.064	−0.056	0.007	−0.017	1
**Higher resilience group (*****n** =* **469; 46.1%)**											
	1	Socioeconomic ladder	1								
	2	Overall worry about COVID-19	−0.044	1							
	3	Family's financial difficulty	−0.186^***^	0.181^***^	1						
	4	Learning problems	−0.073	0.201^**^	0.324^***^	1					
	5	Loneliness	−0.068	0.076	0.137^*^	0.405^***^	1				
	6	Worsening of psychosocial wellbeing	−0.083	0.139^*^	0.139^**^	0.229^**^	0.201^***^	1			
	7	Gender	−0.094^*^	0.219^***^	0.035	0.050	−0.050	0.114^*^	1		
	8	Household size	0.085	−0.030	−0.039	0.097	−0.049	0.007	−0.007	1	
	9	Baseline health status	0.197^***^	0.040	−0.013	−0.035	−0.056	−0.125^*^	−0.045	−0.017	1

^*^p < 0.05; ^**^p < 0.01; ^***^p < 0.001.

**Figure 2:**
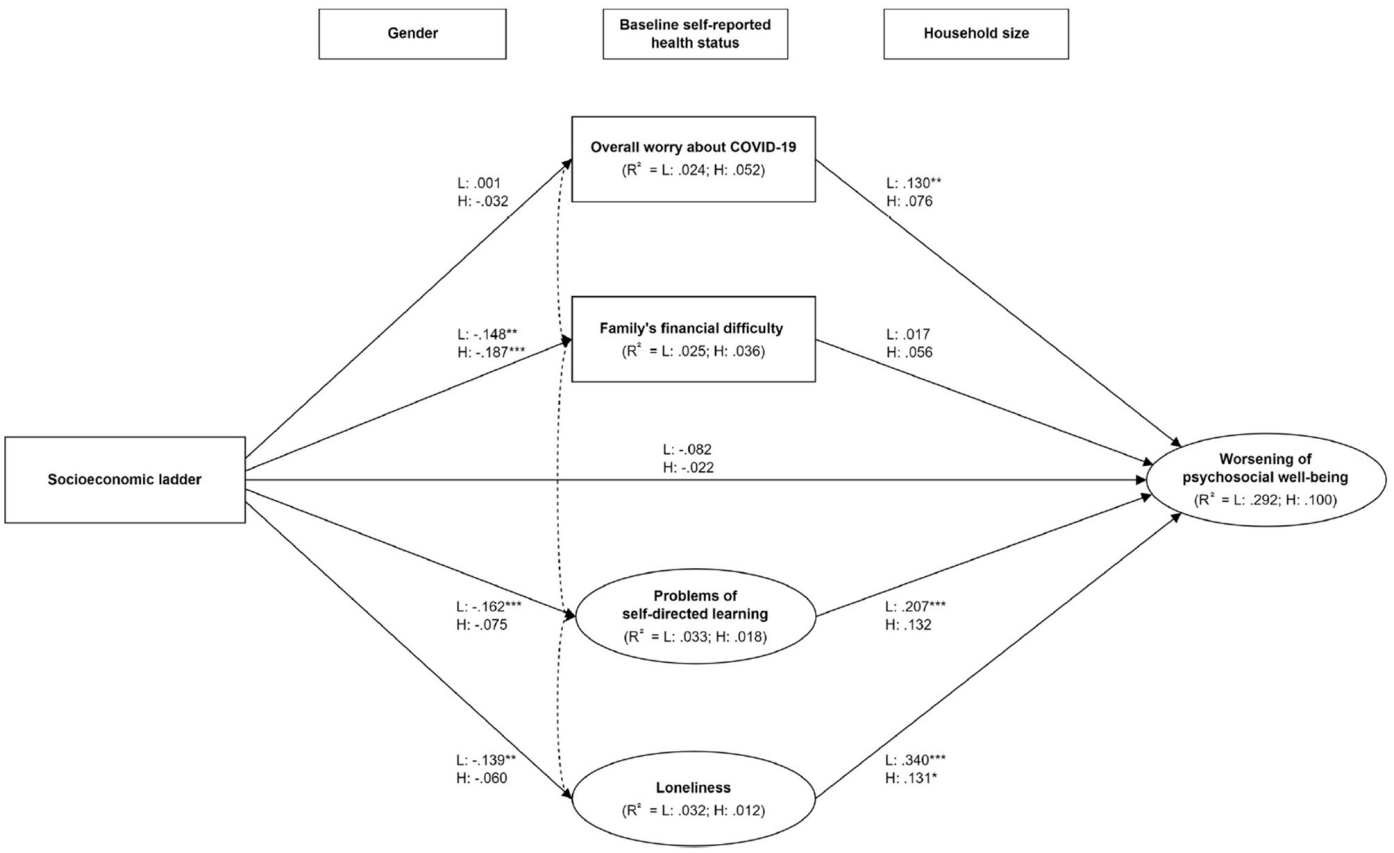
The mediating pathways between socioeconomic position and the worsening of psychosocial wellbeing by resilience levels. L: Lower resilience group; H: Higher resilience group. *p < 0.05; **p < 0.01; ***p < 0.001. Model was adjusted for gender, household size, and baseline self-reported health status. Covariance was specified in each of the possible pairs of mediators. The dotted double arrows between mediators are simplified for better readability.

Results from the multi-group SEM analysis showed that the pattern of socioeconomic patterning and predictors of psychosocial wellbeing in the lower resilience group (*n* = 549) were consistent with that in the overall sample (Figure 2), with stronger effect size in most paths. Nonetheless, the adverse impact of socioeconomic ladder on psychosocial determinants (except for learning problems) and their effects on psychosocial wellbeing were substantially mitigated in the higher resilience group (*n* = 469). The total effect between socioeconomic ladder and the worsening of psychosocial wellbeing was significant in the lower resilience group (β = −0.166 [95% CI = −0.259 – −0.072], *p* < 0.001) but not in the higher resilience group (β = −0.053 [95% CI = −0.159 – 0.050], *p* = 0.322). In addition, the significant χ^2^ difference (change in χ^2^ = 48.703, change in df = 33, *p* = 0.038) between the unconstrained model and structural weight model indicated the difference of models between the lower and higher resilience groups in explaining the paths among socioeconomic ladder, mediators, and the worsening of psychosocial wellbeing. In particular, the indirect effects between socioeconomic ladder and the worsening of psychosocial wellbeing through learning problems (*p* = 0.001) and loneliness (*p* < 0.001) were significant only in the lower resilience group but not in the higher resilience group (*p* = 0.140 and *p* = 0.130, respectively).

## Discussion

The present study is the first to employ SEM to examine the socioeconomic patterning and psychosocial risks of COVID-19-related disrupted social conditions among adolescents of different resilience level in Hong Kong. In general, the worsening of psychosocial wellbeing was strongly patterned across the socioeconomic ladder because of the greater learning problems and loneliness experienced by socioeconomically disadvantaged adolescents during the pandemic. Nonetheless, adolescents of higher resilience have fared better in response to COVID-19 and overcome part of the adverse impact of socioeconomic disadvantage on social conditions and hence their psychosocial wellbeing.

Consistent with the existing literature, our findings supported that adolescents of lower socioeconomic position are more vulnerable to psychosocial distress under the pandemic (9, 10). Given that the outbreaks in Hong Kong are relatively well-controlled with 12,650 cases and 213 deaths by the end of 2021 (32), worries about COVID-19 infection and mortality are not likely explanations for the worsening of psychosocial wellbeing. More plausibly, the stronger psychosocial impact on the socioeconomically disadvantaged might have been resulted from the differential socioeconomic impact of stringent containment measures under the “zero-infection” policy (i.e., preventing imported cases from spreading into the community to maintain zero local infection) in Hong Kong. In particular, the prolonged school closure has posed significant but disproportionate challenges to both their learning experience and psychosocial wellbeing (11, 12, 33). Although distance learning serves as a crucial educational resource and platform during the pandemic, research showed that the shifting from face-to-face to online classes by itself is a psychosocial stressor to students (34). Notably, education disruption due to school closure has resulted in poorer learning gains especially among students from low-income families. Local research also showed that the effectiveness of distance learning was patterned by household income levels (35), whereas, limitations of home environment to support self-directed learning (e.g., disturbance by family members as well as a lack of resources and space) were frequently reported even by the middle class during the pandemic (36). Given the buffering effect of distance learning satisfaction against COVID-19-induced psychosocial stressors (37), it comes as no surprise that the socioeconomically disadvantaged adolescents, who faced greater difficulties and dissatisfaction with distance learning during the pandemic, had poorer adjustment in response to COVID-19 and thus suffer from greater psychosocial distress. Our findings echoed with the above studies that the socioeconomic patterning of the worsening of psychosocial wellbeing was partially mediated through the greater learning problems among the socioeconomically disadvantaged.

In addition to learning problems, the extent of loneliness during the pandemic appeared to explain part of the association between socioeconomic position and psychosocial wellbeing among adolescents. While the stringent social distancing measures imposed by the Hong Kong government during the waves of severe local outbreaks [e.g., school closure, prohibition on group gatherings of more than two/four people in public places and dine-in services at night, and closure of leisure facilities and entertainment premises, etc. (38)] have served their purpose of containing the spread of COVID-19, they also seriously disrupted the social life of adolescents. As an inadvertent consequence of social distancing measures, loneliness is particularly problematic for adolescents due to the criticality of peer support and the formation of social identity during their developmental stage (39), which in turn exacerbated the psychosocial impacts of COVID-19 (13, 40). The greater susceptibility to loneliness among socioeconomically disadvantaged adolescents could possibly be attributed to the fewer quality time with and perceived support from family and friends when confined at home (16), inadequate private space for social activities (41), higher vulnerability to the harmful use of social media (42), and greater difficulty developing new hobbies to distract themselves from loneliness (43). These speculations accord with the fundamental cause theory that people of lower social status lack capabilities and resources, such as money, space, social capital, digital literacy, and other health and social advantages, to overcome stressors and improve psychosocial wellbeing (44, 45).

Our findings have provided insights on several potential entry points for interventions to buffer the psychosocial impact of further outbreaks and school closures on adolescents. To facilitate self-directed learning, feasible approaches include providing students with broadband internet access and technical support for distance learning, interactive tutorials, and counseling services for need assessment (34, 37), whereas to ease loneliness, addressing maladaptive social cognition as well as enhancing students' emotional awareness and reconciliation *via* improvement on inter-personal and intra-personal skills may be possible options (46, 47). In addition, deep listening and non-judgmental acceptance by parents are crucial to identify emotional issues of adolescents at an early stage (48). Besides, our results on the moderating effect of resilience also highlighted the criticality of resilience building among adolescents, especially after schools re-open as resilience-focused interventions are commonly school-based (49). As suggested by a recent systematic review, schools may be the best setting to develop resilience of students, especially the most disadvantaged group, by providing multiple types of resources including access to material resources and supportive relationships, experience of power and control, social justice, and social cohesion with others, as well as development of desirable personal identity and adherence to cultural traditions (50). In light of this, educators should work with social workers and psychologists to review the current school-based psychosocial support programs, and consider incorporating positive psychology and cognitive behavioral therapy-based approaches into resilience-focused interventions (49). From a more upstream perspective, while the stringent social distancing measures and school closure may be able to protect students from COVID-19 infections, the tremendous cost of these measures on a wide array of social determinants of health should not be overlooked. Previous research has pointed out that the “zero-infection” approach is highly prone to neglect social and health inequities, which is neither ethical nor feasible in the long run (51). Therefore, in addition to allocating extra resources to support the disadvantaged groups, policy makers should carefully consider the impact on social determinants of health when devising a long-term response to COVID-19 so as to balance disease containment with the psychosocial wellbeing, developmental opportunities, and equity of adolescents.

There are several limitations of the present study. First, the cross-sectional design of our survey could not establish temporality for causal inferences. Second, we adopted purposive sampling of schools due to the difficulty in random sampling under the pandemic. Although the selected schools were not a statistically representative sample, we recruited schools of diverse socioeconomic background with considerations for a balanced gender ratio to maximize the qualitative generalizability of our sample. Third, as the assessment of key variables were based on self-reported responses to survey, our results may be subject to recall bias and social desirability bias. Fourth, the goodness-of-fit of the SEM model may be affected by the inclusion of single-item ordinal mediators (i.e., overall worry about COVID-19 and family's financial difficulty). To this end, we have replicated the SEM analysis without these two mediators and the model fit remained satisfactory with χ^2^ (df = 86, *N* = 1018) = 301.892, *p* < 0.001, RMSEA = 0.050, RMR = 0.033 CFI = 0.957, IFI = 0.958, TLI = 0.940, and AGFI = 0.944. Last, despite adjustment for gender, household size, and baseline health status, residual confounding is possible due to the unavailability of data on history of mental health disorders, lifestyle behaviors, and healthcare access.

## Conclusion

Adolescents of lower socioeconomic position, especially those with a lower level of resilience, were at higher risk of experiencing psychosocial distress during the COVID-19 pandemic because of greater learning problems and loneliness under the differential socioeconomic impact of stringent social distancing measures in Hong Kong. In addition to providing distance learning and social support, evidence-based strategies to build up resilience among adolescents are crucial to buffer against the adverse socioeconomic and psychosocial impacts of the pandemic.

## Data availability statement

The raw data supporting the conclusions of this article will be made available by the authors, without undue reservation.

## Ethics statement

The studies involving human participants were reviewed and approved by the Survey and Behavioral Research Ethics Committee of the Chinese University of Hong Kong. Written informed consent to participate in this study was provided by the participants' legal guardian/next of kin.

## Author contributions

GKKC contributed to literature search, study design, data analysis, result interpretation, and the write-up of the manuscript. YHC and SMC were responsible for study design, data curation, and result interpretation. TSKL contributed to data collection, coordination, and data analysis. JKC was responsible for study design and provided substantial statistical advice on the analyses. HW and RYNC oversaw the project as the co-Principal Investigators, contributed to study design, and result interpretation. ESCH was responsible for data collection, data analysis, and result interpretation. All authors critically appraised and approved the manuscript.
