# Supplementary material for: Socioeconomic inequality in the worsening of psychosocial wellbeing via disrupted social conditions during COVID-19 among adolescents in Hong Kong: self-resilience matters
Source: Front Public Health. 2023 Apr 26;11:1136744. doi: 10.3389/fpubh.2023.1136744 (PMC10169685; doi:10.3389/fpubh.2023.1136744)
Supplement: Supplementary file 1 [file Table_1.DOCX]

The socioeconomic background of interested schools, supplemented with their academic intake, was predicted by the Program for International Student Assessment (PISA) index of economic, social, and cultural status (ESCS) obtained in previous cycles, which was derived from parental education, highest parental occupation, and home possessions. Eventually, 12 secondary schools of different PISA index of ESCS were selected to participate in the survey. To triangulate the socioeconomic background of the schools, we further analyzed respondents’ self-reported socioeconomic positions on parental education levels and the perceived socioeconomic ladder to generate a school-level socioeconomic index using principal component analysis. Hence, the participating schools were classified into five levels according to the resultant school-level socioeconomic index

| **Supplementary Table 1: Socioeconomic background of the participating schools** | | |
| --- | --- | --- |
| School | School-level socioeconomic index | Socioeconomic level |
| 1 | -0.469 | Low |
| 2 | -0.867 | Low |
| 3 | -0.264 | Mid-Low |
| 4 | -0.371 | Mid-Low |
| 5 | -0.091 | Middle |
| 6 | -0.26 | Middle |
| 7 | -0.239 | Middle |
| 8 | -0.126 | Middle |
| 9 | 0.021 | Mid-High |
| 10 | 0.037 | Mid-High |
| 11 | 0.515 | High |
| 12 | 1.132 | High |
